# The influence of nationwide COVID-19 lockdown on the functional impairment and long-term survival of dependent people for carrying out basic activities of daily living in a neighborhood of the city of Madrid, Spain: Orcasitas Cohort Longitudinal Study

**DOI:** 10.3389/fpubh.2024.1385058

**Published:** 2024-07-09

**Authors:** Vicente Martín Moreno, María Inmaculada Martínez Sanz, Miriam Fernández Gallardo, Amanda Martín Fernández, María Palma Benítez Calderón, Helena Alonso Samperiz, Elena Pérez Rico, Laura Calderón Jiménez, Sara Guerra Maroto, Elena Sánchez Rodríguez, Eva Sevillano Fuentes, Irene Sánchez González, Miguel Recuero Vázquez, Julia Herranz Hernando, Irene León Saiz

**Affiliations:** ^1^Orcasitas Health Care Center and i+12 Research Institute of the Doce de Octubre Hospital, Grupo de Investigación sobre Dependencia en Orcasitas (GIDO Collaborative Group), Madrid, Spain; ^2^Orcasitas Health Care Center, Grupo de Investigación sobre Dependencia en Orcasitas (GIDO Collaborative Group), Madrid, Spain; ^3^Polibea Concert, Grupo de Investigación sobre Dependencia en Orcasitas (GIDO Collaborative Group), Madrid, Spain; ^4^Orcasitas Health Care Center, Grupo de Investigación sobre Dependencia en Orcasitas (GIDO Collaborative Group), Nursing Home Care Unit of the Center, Madrid, Spain

**Keywords:** COVID-19, basic activities of daily living, Barthel, functional impairment, social inequalities, vulnerability, frailty, salutogenesis

## Abstract

**Background:**

Prolonged confinement can lead to personal deterioration at various levels. We studied this phenomenon during the nationwide COVID-19 lockdown in a functionally dependent population of the Orcasitas neighborhood of Madrid, Spain, by measuring their ability to perform basic activities of daily living and their mortality rate.

**Methods:**

A total of 127 patients were included in the Orcasitas cohort. Of this cohort, 78.7% were female, 21.3% were male, and their mean age was 86 years. All participants had a Barthel index of ≤ 60. Changes from pre- to post-confinement and 3 years afterward were analyzed, and the effect of these changes on survival was assessed (2020–2023).

**Results:**

The post-confinement functional assessment showed significant improvement in independence over pre-confinement for both the Barthel score (*t* = −5.823; *p* < 0.001) and the classification level (*z* = −2.988; *p* < 0.003). This improvement progressively disappeared in the following 3 years, and 40.9% of the patients in this cohort died during this period. These outcomes were associated with the Barthel index (*z* = −3.646; *p* < 0.001) and the level of dependence (hazard ratio 2.227; CI 1.514–3.276). Higher mortality was observed among men (HR 1.745; CI 1.045–2.915) and those with severe dependence (HR 2.169; CI 1.469–3.201). Setting the cutoff point of the Barthel index at 40 provided the best detection of the risk of death associated with dependence.

**Conclusions:**

Home confinement and the risk of death due to the COVID-19 pandemic awakened a form of resilience in the face of adversity among the population of functionally dependent adults. The Barthel index is a good predictor of medium- and long-term mortality and is a useful method for detecting populations at risk in health planning. A cutoff score of 40 is useful for this purpose. To a certain extent, the non-institutionalized dependent population is an invisible population. Future studies should analyze the causes of the high mortality observed.

## Introduction

The COVID-19 pandemic brought about changes in personal and social habits unprecedented in this century and altered the social and health balance of affected countries. In Spain, nationwide lockdown was decreed on March 15, 2020. This measure slowed the spread of the pandemic but had an impact on people, especially dependents (1).

Dependence is a vulnerability criterion that increases the risk of hospitalization and death (2). Although confinement affected all population groups, dependent people were among the most sensitive to this situation since their dependence is associated with greater age and comorbidities, which were risk factors for morbidity and mortality due to COVID-19 (3–5)^.^ Further, the risk of contagion is probably greater among dependent populations, especially those institutionalized, due to the need for interaction with others for care (6).

Apart from the COVID-19 pandemic, dependency is an emerging socio-health problem that requires multiple socio-health resources, with a progressively increasing cost, and with two models of care, depending on whether the dependent persons reside at home or are institutionalized in nursing homes. To maintain equity in the care of both groups, it is necessary to carry out adequate healthcare planning and evaluate the actions carried out (7). The 20th Report of the State Observatory on Dependency (8), February 2020, states that 3% of the Spanish population needs support to perform basic activities of daily living (ADL), most of this population being female. At the healthcare level, care for dependency falls on primary care nurses, the main clinical manager of this multidisciplinary problem (9).

However, dependency is not a standalone issue and requires a broader approach. Chronicity, complexity, and dependency form an increasingly prevalent interrelated triad in our society (10), contributing to the creation of vulnerable populations and social inequalities (11). Thus, in a pandemic, with the added factor of confinement, new conditions and rules of the game were created, which need to be analyzed to design strategies that will enable us to deal with these situations in the future.

The new rules of social and personal functioning and supervision, for which no one had an adaptation period, included the elimination of visits by relatives to those not strictly necessary, leaving food at the door, the loss of attention in day centers, and the suspension of home care performed by hired caregivers. These changes aggravated the loneliness of the dependent adults and forced them to perform functions and responsibilities that they had not before. All the above was added to the fear generated by the media bombardment, showing a high mortality rate among people like them. All the above factors impact the physical and mental health of people, which requires further analysis (12, 13).

Although previous studies have examined institutionalized dependent populations (14–16) or involved research before and after hospital discharge (17, 18), generally in association with patient interventions (19), there have been fewer longitudinal studies of non-institutionalized dependent populations in the context of the COVID-19 pandemic. A 2019 study addressed the complexity of these factors and their interrelationships (20). The current study aims to analyze whether the nationwide lockdown, which was declared under a state of alarm by the Government of Spain, conditioned short- and long-term changes in the functional dependence of the dependent population of the Orcasitas neighborhood of Madrid (Spain), and to quantify the relationships of these changes with sociodemographic variables and survival at 3 years.

## Materials and methods

### Design

A longitudinal descriptive study was conducted in the Orcasitas neighborhood of the city of Madrid (Spain) between June 2020 and August 2023 on the entire functionally dependent population assigned to the Orcasitas Healthcare Center that was registered in the e-SOAP application of the Community of Madrid. One hundred and fifty patients were included in the Orcasitas cohort. The nationwide lockdown began in Spain on March 15, 2020. Madrid moved to phase 1 of the de-escalation on May 18, 2020, and the state of alarm ended on June 21, 2020. In the de-escalation phase 1, people were allowed to leave for non-compulsory activities within time limits.

The Orcasitas Healthcare Center is a primary care center that serves the basic health area of Orcasitas, a neighborhood in Madrid (Spain) with a population of 22,452 people. It provides health services in person at the center, by telematic means, and at the patient's home, as required.

### Participants

Inclusion criteria were: participants needed to be 65 years or older, have a diagnosis of functional dependence to perform basic activities of daily living (ADL), and have a previous Barthel score in the last year. The exclusion criteria were: 1: Not belonging to the basic health area. 2: Not being in the usual home during the study period (hospital or nursing home admission, older adults living in their children's homes). 3: Not having a previous Barthel score in the last year. 4: Not being able to respond autonomously or through a trained usual caregiver to the questionnaires used. 5: Having a diagnosis of a terminal illness. Five patients refused to participate in the study. And 18 were excluded: not located (*n* = 1), being in another home (*n* = 2), admission to a nursing home (*n* = 4), admission to hospital (*n* = 2), death before the start of the study (*n* = 4), not dependent due to error in previous Barthel score (*n* = 1), and not having a previous Barthel score (*n* = 4). Finally, 127 patients participated.

This study was approved by the Center Local Research Commission of the Primary Care Management of Madrid (reference number 16/20-C-Bis) on June 29, 2020. The Ethics Committee of the Hospital 12 de Octubre endorsed this approval as sufficient in resolution CEIm 23/501 dated September 26, 2023.

### Data collection

Functional dependence in ADL was assessed using the Barthel index, according to the internationally recognized classification (21) used as a criterion in the Spanish National Health System Primary Care dependence protocol (22), with four levels of dependence: mild (Barthel 60), moderate (Barthel 40–55), severe (Barthel 20–35), and total (Barthel < 20). A second cutoff point was also established at Barthel 40, with two groups, severe dependents (Barthel < 40) and moderate dependents (Barthel 40–60) (23). The pre-pandemic baseline Barthel, Barthel in June 2020, and Barthel in 2023 were recorded.

The Barthel assessment was performed by a single blind, with the nursing staff unaware of the pre-confinement value. At the same visit, the variables that could be studied were obtained using a previously validated questionnaire that collected age, sex, marital status, and educational level. The questionnaire was validated through a pilot study in a population over 80 years of age, dependent and non-dependent, in the Orcasitas neighborhood. It consisted of 48 questions, mostly closed and generally dichotomous. To avoid errors in the way the questionnaire was administered, some questions included explanatory notes on the correct way to ask the question, with the double objective of avoiding bias associated with the interviewer, and the way the question was asked would condition a different response in the interviewee. No question required modifications in its wording to facilitate its comprehension in the pilot study.

### Definitions of covariates

The clinical variables, body mass index (BMI), number of chronic diseases, and whether the patient was polymedicated (cutoff point five active components), were collected through the AP-Madrid application of the Health System. The Individual Health Card (IHC) application was used to obtain the number of persons aged 65 years or over registered at the Orcasitas Health Center by age group and the economic income data.

The IHC and AP-Madrid applications classified the population over 65 years according to their level of income in two groups: (1) income above 11,200 euros/year; and (2) income below 11,200 euros/year. This cutoff point was used in this study for the analyses associated with this variable. With respect to educational level and given the high illiteracy rates in the Orcasitas neighborhood, two categories were established: having education and not having education (illiterate, knowing how to read and write, but not having studied and not having completed primary education).

The housing situation of the dependent persons was classified into three groups: (1) they live independently, either alone or with a partner; (2) they live with their children; and (3) they live with other people who are neither children nor a partner.

Age was assessed in the statistical analyses as a quantitative variable, but functional dependents were also classified into three age groups: (1) age < 80; (2) age between 80 and 89; and (3) age equal to or more than 90 years.

The date of death of each functionally dependent person during the study period (June 2020 to June 2023) was recorded, obtaining the data from the Health System's IHC application.

### Data analysis

The analysis of the collected variables was performed with the statistical package SPSS 18.0. First, a descriptive analysis of the sample was performed. Continuous variables were described as mean and standard error, and categorical variables as absolute numbers and/or proportions. The normality of the data distribution was tested using the Shapiro-Wilk test for each variable. When the outcome variable did not follow a normal distribution, ANOVA was performed with Levene's homogeneity test, applying Welch's correction as a robust test of equality of means when the sample size was <30. Differences between continuous variables were analyzed using the Student's *t*-test or the Mann–Whitney *U*-test, and differences between categorical variables were analyzed using the chi-square test. Before-after differences in the Barthel index were analyzed using the Wilcoxon signed-rank test. The probability of occurrence of an event was analyzed by the odds ratio (OR).

ADL dependence was analyzed according to the original Barthel index clusters and was also dichotomized into two categories: “moderate,” which included the categories mild (Barthel 60) and moderate (Barthel 40–55); and “severe,” which included the categories severe (Barthel 20–35) and total (Barthel < 20), in both univariate and multivariate analysis.

Mortality information was obtained for all patients during the 3 years of follow-up. Survival curves for levels on the Barthel index before and after the nationwide COVID-19 lockdown and for each Barthel index item were estimated using the Kaplan–Meier method. Comparisons of the curves were made using the log-rank test.

Univariate analysis was performed to identify the variables associated with the long-term survival of the people included in this cohort and the subgroup of dependent people who showed changes in the Barthel index score during the nationwide COVID-19 lockdown. Covariates that were significant in the bivariate analysis were introduced into the multivariate regression model. The resulting model was summarized by the estimated coefficients, *p*-values, and hazard ratios (HR) with their 95% confidence intervals. Crude and adjusted hazard ratios (HR) and confidence intervals (95% CI) were calculated using Cox proportional regression models.

Using Cox regression, adjusted for sex, educational level, and income level, survival in June 2021 and June 2023 was analyzed according to the level of functional dependence, disaggregated for each Barthel index level. Statistical significance was considered *p* < 0.05.

## Results

### Demographic characteristics

The mean age of the population of the Orcasitas cohort was 86.6 years, with 21.3% of the participants being male, generally married (55.6%; *n* = 15), and 78.7% female, mostly widowed (71%; *n* = 71). A large majority (87.4%) reported having insufficient education (illiterate, has no schooling but can read and write; or having incomplete primary education). The sociodemographic and health variables are presented in Table 1. According to the analysis of variance, no differences were observed between the sexes in terms of the score obtained on the Barthel index, BMI, educational level, or income level (Table 2).

**Table 1:** Clinical and sociodemographic data of the Orcasitas cohort in June 2020 and analysis of their relationship with survival in June 2021.

**Orcasitas cohort: survival in June 2021**			
**Variable**	**Data**	**Statistical**	**Significance**
**Survival**, ***n*** **(%)**			
Alive	102 (80.31%)	–	–
Deceased	25 (19.69%)		
**Sex**, ***n*** **(%)**			
Male	27 (21.3%)	χ^2^ = 0.140	*p* = 0.709
Woman	100 (78.7%)		
**Clinical variables**			
Age (mean ± SD); range 66–102 years	86.6 ± 6.3	*z* = −0.046	*p* = 0.964
BMI (mean ± SD)	28.58 ± 4.70	*z*: −0.238	*p* = 0.812
Barthel pre-pandemic (mean ± SD):	43.28 ± 19.01	*z* = −2.644	*p* = 0.008
Barthel post-pandemic (mean ± SD):	54.41 ± 26.11	*z* = −2.647	*p* = 0.008
**Marital status**^a^, ***n*** **(%)**			
Married	39 (30.7%)	χ^2^ = 0.410^a^	*p* = 0.522
Not married^b^	88 (69.3%)		
**Education level**^c^, ***n*** **(%)**			
Insufficient^d^ (without education)	111 (88.1%)	χ^2^ = 7.704^b^	*p* = 0.006
Has education^e^	15 (11.9%)		
**Income level**, ***n*** **(%)**			
<11,200 euros/year	60 (47.24%)	χ^2^ = 10.327	*p* = 0.001
≥11,200 euros/year	67 (52.76%)		
**Chronicity burden**, ***n*** **(%)**			
≥5 chronic diseases e-SOAP^f^	34 (27%)	χ^2^ = 1.352	*p* = 0.245
<5 chronic diseases e-SOAP	92 (73%)		
**Polymedication**^g^, ***n*** **(%)**			
Yes	115 (90.6%)	χ^2^ = 4.971	*p* = 0.026
No	11 (8.7%)		

^a^Analysis of the differences between being married and having other marital statuses.

^b^Not married: includes widowed, separated, divorced, and single people.

^c^Analysis of the differences between having and not having an education.

^d^Includes: illiterate, has no schooling but can read and write, and not finished primary education.

^e^Includes having some type of education (primary, graduated, higher education).

^f^e-SOAP: IT tool that manages the primary care scorecard.

^g^Polymedicated: cutoff point 5 or more drugs or active substances.

**Table 2:** Analysis of the differences between the socioeconomic and health variables based on sex and survival at 1 and 3 years.

**contrast between variables in relation to sex**					
**Variable**		**Sex**	**Data**	**Statistical**	**Significance**
BMI (mean ± SD)		Male	27.43 ± 3.684	*z* = −1.503	*p* = 0.133
		Female	28.90 ± 4.914		
Age (mean ± SD)		Male	87.19 ± 5.962	*z* = −0.345	*p* = 0.730
		Female	86.48 ± 6.414		
Dependency *n* (%)	Severe	Male	8 (21.05%)
		Female	30 (78.95%)	χ^2^ = 0.001	*p* = 0.970
	Moderate	Male	19 (21.35%)		
		Female	70 (78.65%)
Education level^a^*n* (%)	Insufficient (without education)	Male	23 (20.72%)
		Female	88 (79.28%)	χ^2^ = 0.277	*p* = 0.598
	Has education	Male	4 (26.67%)		
		Female	11 (73.33%)
Income level *n* (%)	Income ≥ 11,200 euros/year	Male	14 (20.89%)
		Female	53 (79.11%)	χ^2^ = 0.011	*p* = 0.916
	Income < 11,200 euros/year	Male	13 (21.67%)		
		Female	47 (78.33%)
Survival June 2021	Alive	Male	21 (77.78%)	χ^2^ = 0.140 HR 0.864^b^ HR 1.039^c^	*p* = 0.709 CI 0.383–1.948 CI 0.831–1.299
		Female	81 (81%)		
	Deceased	Male	6 (22.22%)
		Female	19 (19%)
Survival June 2023	Alive	Male	10 (37.03%)	χ^2^ = 6.87 HR 1.745^b^ HR 1.395^c^	*p* = 0.009 CI 1.045–2.915 CI 1.026–1.896
		Female	65 (65%)		
	Deceased	Male	17 (62.96%)
		Female	35 (35%)

^a^Analysis of the differences between people without education (illiterate: has no schooling but can read and write, not finished primary education) and having some type of education (primary, graduated, higher education).

^b^HR, hazard ratio, univariate regression.

^c^HR, multivariate regression, adjusted for educational level, economic level, and Barthel level.

CI, confidence interval.

About the population of the Orcasitas neighborhood (Madrid), and within the group of persons over 65 years of age, the IHC application of the Health System of the community of Madrid established three population groups: patients aged 65–74 years (1,808 persons); patients aged 75–79 years (703 persons); and patients aged 80 years or more (1,797 persons). In the dependent population, among the non-dependent population, the group of people over 80 years of age was highly represented. Out of a total population of 22,452 people in the Orcasitas neighborhood in June 2020, the group of people over 80 years of age represented 8%, and the group of people over 65 represented 19.2%. The group of people under 14 years of age (3,248 people) assigned to this health center represented 14.5% of the population of this neighborhood. The population with functional dependency in the Orcasitas neighborhood represented 7.1% of the population over 80 years of age and 3.1% of the population over 65 years of age. The aging index ([population over 65 years old/population under 14 years old] × 100) was 1.33.

### Changes in the level of functional dependence due to nationwide COVID-19 lockdown

Before confinement, 15% (*n* = 19) of the persons in this cohort had total dependence (Barthel < 20), 15% (*n* = 19) severe dependence (Barthel 20–35), 36.2% (*n* = 46) moderate dependence (Barthel 40–55), and 33.9% mild dependence (Barthel 60). The mean Barthel index scores before confinement were 43.28 ± 19.017.

After confinement, 11% (*n* = 14) had total dependence, 15% (*n* = 19) had severe dependence, 21.3% (*n* = 27) had moderate dependence, and 9.4% (*n* = 12) had mild dependence. Approximately 43.3% (*n* = 55) of those who were dependent pre-confinement ceased to be dependent after confinement by improving their Barthel score and surpassing the cutoff point of 60. The mean Barthel index scores after confinement were 54.41 ± 26.115.

**Figure 1:**
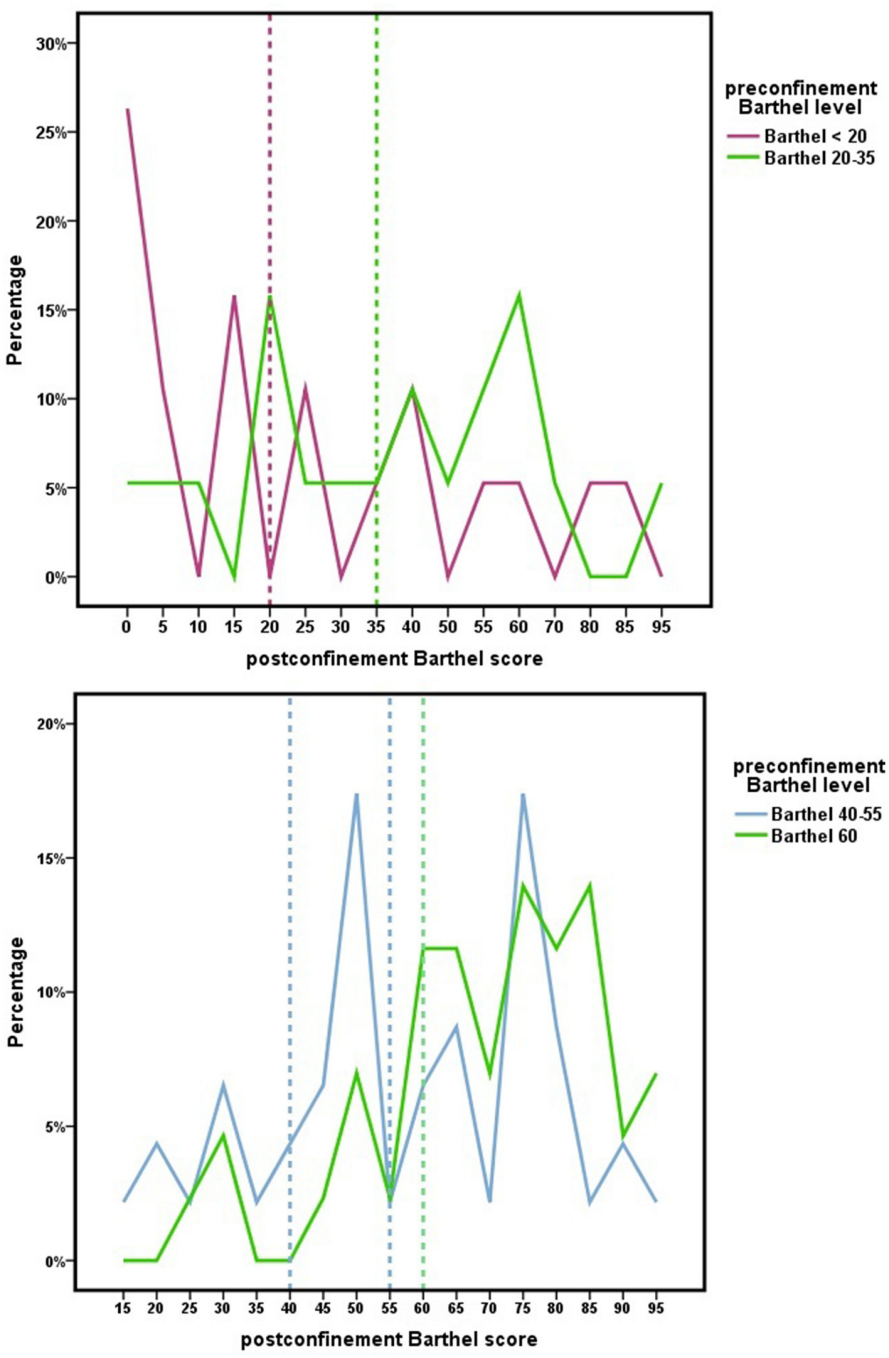
Changes in the score in the Barthel index because of the nationwide COVID-19 lockdown for each level in the Barthel index. Dependency is established for a Barthel index score of 60 or less. After confinement, the scores achieved are reflected, indicating a Barthel index score > 60 that the person no longer meets dependency criteria according to the National Health System protocol.

A significant improvement (*t* = −5.823; *p* < 0.001) in functional dependence to perform ADLs during national COVID-19 confinement was observed in 62.2% of patients in the Orcasitas cohort. Twenty-six percentage of the dependent persons worsened their baseline Barthel index score. The improvement in the Barthel index score was reflected in the level of dependency (χ^2^ = 28.710; *p* < 0.001), a result shown in Figure 1. This figure combines the two most widely used dependency classification models using the Barthel index. The decision to combine both models in the same figure allowed us to reflect the impact of home confinement due to the COVID-19 pandemic on the ability to perform basic activities of daily living for each level of dependency of the classic Barthel scale. It also helped to investigate the second classification model analyzed in this study with two levels, severe (Barthel < 20 and Barthel 20–35) and moderate (Barthel 40–55 and Barthel 60).

**Figure 2:**
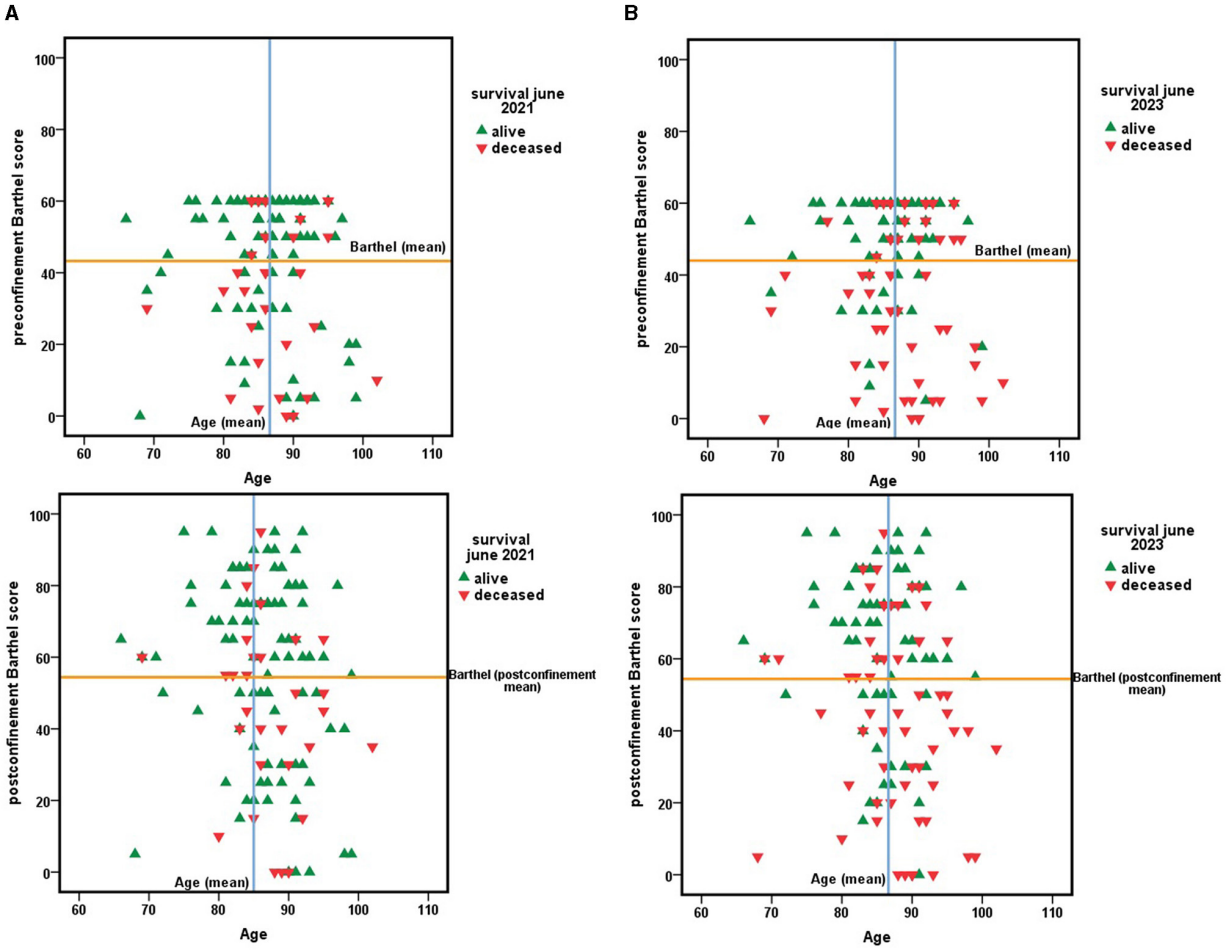
**(A)** Survival 1 year after the end of the nationwide COVID-19 lockdown depending on age and functional dependency, estimated by the Barthel index score at two time cutpoints, before March 2020, the start date of confinement, and in June 2020, the end date of confinement in Spain. **(B)** Survival 3 years after the end of the nationwide COVID-19 lockdown. A function of age and functional dependency, estimated by the Barthel index score at two time cutpoints, before March 2020, the start date of confinement, and June 2020, the end date of confinement in Spain.

Figure 2 includes the age factor as the central axis on which to observe the changes in functional capacity during confinement, estimated by the Barthel scale score, and the effect of these changes, or their absence, in terms of survival at 1 and 3 years.

A total of 43.3% (*n* = 55) of the dependent persons in this cohort ceased to meet the inclusion criteria of the dependency protocol after the nationwide COVID-19 lockdown by improving their Barthel score and exceeding the cutoff point of 60. Three years later (June 2023), 56.4% (*n* = 31) of these functionally dependent persons who ceased to be functionally dependent during nationwide COVID-19 lockdown remained non-dependent, while, regarding those who improved their functional abilities, 3 years later, 29.9% (*n* = 38) maintained such improvement.

These results, observed for any level of baseline dependence (Figure 1; Table 3), allowed 10.5% of people with total or severe dependence to improve their dependence to the point of no longer being dependent. This percentage was much higher among persons with moderate dependence (45.7%) or mild dependence (69.8%). For others, however, confinement led to a deterioration in their baseline state, with 11.8% increasing their level of dependence by one level and 3.1% by two levels.

**Table 3:** Changes observed in June 2020 in the score and classification in the Barthel index because of the nationwide COVID-19 lockdown decreed by the Government of Spain (March-May 2020) and assessment of the changes observed after 3 years (June 2023).

**Changes in the scores and levels on the Barthel index during the nationwide COVID-19 lockdown**		
**Barthel score**	**Barthel postconfinement**^a^ **(June 2020**, ***n*** = **127)**	**Barthel follow-up**^b^ **(June 2023**, ***n*** = **55)**
	***n*** **(%)**	***n*** **(%)**
**Barthel score change compared to pre-confinement**		
- Stayed at baseline value	15 (11.8%)	4 (7.3%)
- Improved over baseline	79 (62.2%)	38 (69.1%)
- Worsened from baseline	33 (26%)	13 (23.6%)
**Assigned level on the Barthel index**	**Barthel postlockdown** ^a^ **(June 2020)**	**Barthel follow-up** ^b^ **(June 2023)**
	***n*** **(%)**	***n*** **(%)**
**Barthel level change compared to pre-confinement**		
- Stayed at baseline value	35 (27.6%)	10 (18.2%)
- Improved over baseline by one level	11 (8.7%)	7 (12.7%)
- Improved over baseline by two levels	6 (4.7%)	3 (5,5%)
- Improved over baseline by three levels	1 (0,8%)	0
- Non-dependent	55 (43.3%)^c^	31 (56.3%)^d^
- Worsened from baseline by one level	15 (11.8%)	3 (5.5%)
- Worsened from baseline by two levels	4 (3.1%)	1 (1.8%)
**Barthel level change compared to pre-confinement, by levels**		
**1. Level 1. Barthel under 20:**		
- Stayed at baseline value	10 (52.6%)	1 (11.1%)
- Improved over baseline by one level	3 (15.8%)	4 (44.5%)
- Improved over baseline by two levels	3 (15.8%)	3 (33.3%)
- Improves the baseline value three levels	1 (5.3%)	0
- Non-dependent	2 (10.5%)^c^	1 (11.1%)^d^
**2. Level 2. Barthel 20–35:**		
- Stayed at baseline value	6 (31.6%)	2 (40%)
- Improved over baseline by one level	5 (26.3%)	1 (20%)
- Improved over baseline by two levels	3 (15.8%)	0
- Non-dependent	2 (10.5%)^c^	2 (40%)^d^
- Worsened from baseline one level	3 (15.8%)	0
**3. Level 3. Barthel 40–55:**		
- Stayed at baseline value	14 (30.4%)	5 (20.8%)
- Increased over baseline by one level	3 (6.5%)	2 (8.3%)
- Non-dependent	21 (45.7%)^c^	13 (54.2%)^d^
- Worsened from baseline one level	7 (15.2%)	3 (12.5%)
- Worsened from baseline by two levels	1 (2.2%)	1 (4.2%)
**4. Level 4. Barthel 60:**		
- Stayed at baseline value	5 (11.6%)	2 (11.8%)
- Non-dependent	30 (69.8%)^c^	15 (88.2%)^d^
- Worsened from baseline one level	5 (11.6%)	0
- Worsened from baseline by two levels	3 (7%)	0

^a^difference between pre-confinement and post-confinement Barthel.

^b^the difference between post-confinement Barthel (June 2020) and Barthel in June 2023.

^c^Number of persons (%) who cease to be functionally dependent during confinement during the COVID-19 pandemic.

^d^Number of persons (%) who ceased to be functionally dependent after the COVID-19 pandemic confinement and maintain functional independence as of June 2023.

We also observed that the percentage of dependent persons who improved their abilities was higher among persons who were previously less dependent (Barthel 60). And this result was observed both against people with total dependence (Barthel < 20; χ^2^ = 9.030; *p* < 0.01) and against people with moderate dependence (Barthel 40–55; χ^2^ = 4.810; *p* < 0.05) but not against people with severe dependence (Barthel 20–35; χ^2^ = 3.500; *p* = NS).

Improving or worsening the Barthel score did not imply changing the level on the scale, with 27.6% maintaining the same level and 72.4% modifying their level, including patients with total dependence (Table 3). Among the group that improved their functional capacity during confinement, 48.1% (38) maintained this improvement at 3 years, representing 69.1% of the cohort in 2023.

The changes in functional capacity during confinement reflected in the Barthel index score performed in June 2020 were not associated with greater or lesser comorbidity (*z* = −0.221; *p* = 0.825), nor with greater or lesser drug consumption in relation to that comorbidity (*z* = −0.386; *p* = 0.700). When the subgroups were analyzed, these results were maintained, and no association was observed between improving the Barthel index score during confinement with disease burden (*z* = −0.386; *p* = 0.699) or with being polymedicated (*z* = −1.329; *p* = 0.184). Among the persons whose functional capacity worsened during confinement, none was a polymedicated patient, and this worsening was not associated with disease burden (*z* = −0.204; *p* = 0.222).

**Figure 3:**
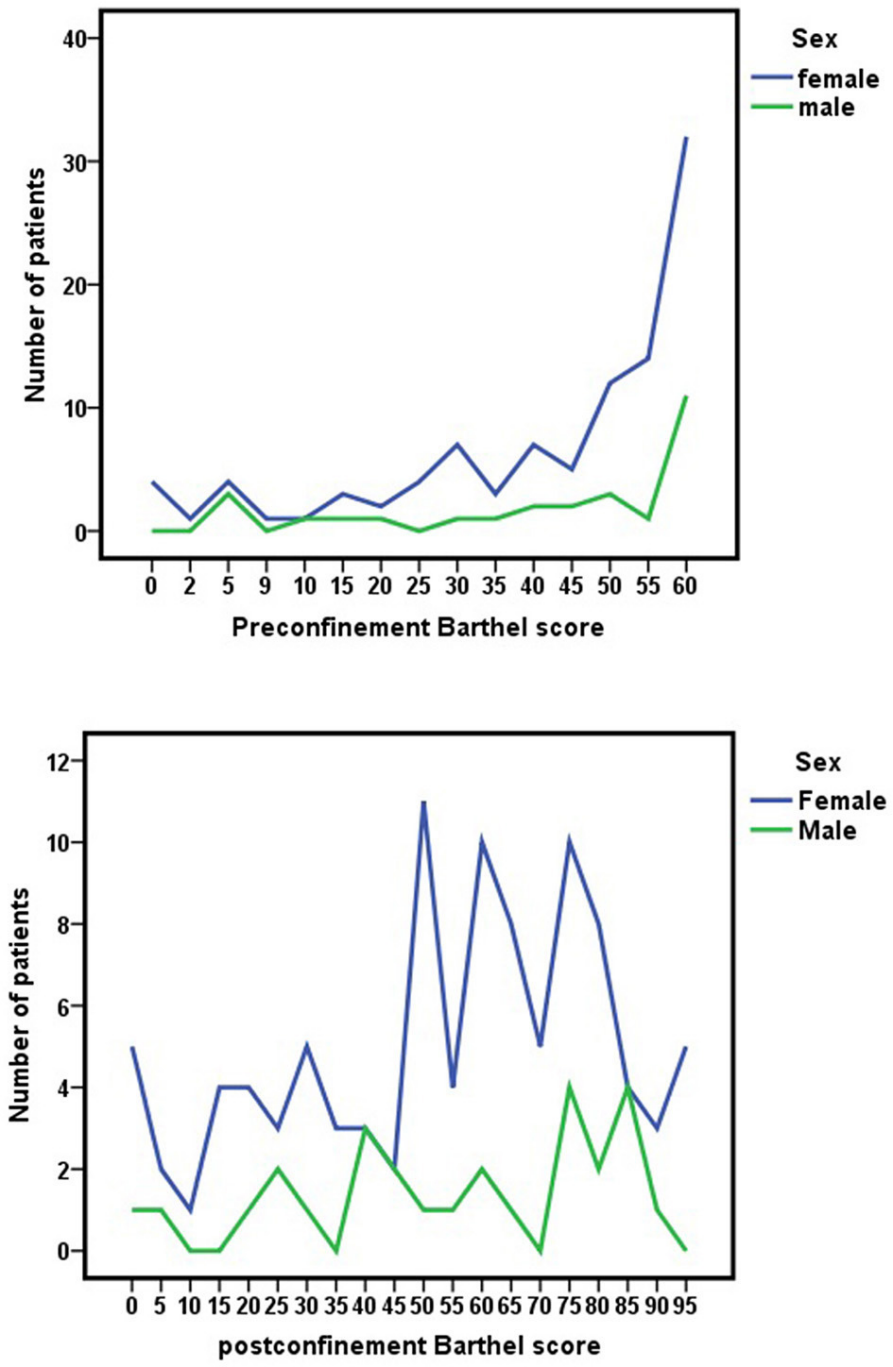
Pre-lockdown Barthel index scores (before March 15, 2020) and changes by the nationwide COVID-19 lockdown in Barthel scores (June 2020) for each sex.

**Figure 4:**
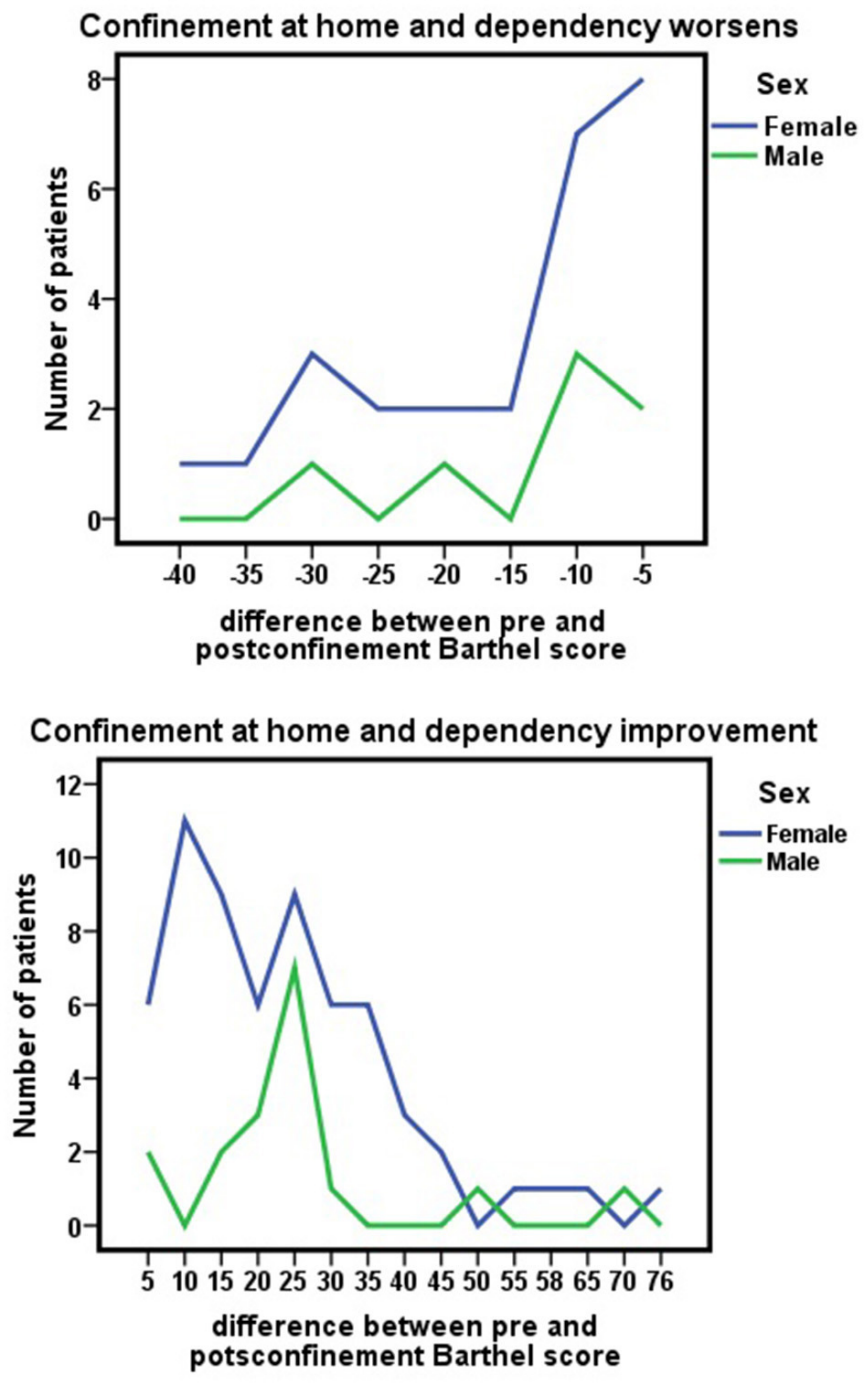
Improvement or worsening of Barthel scores due to nationwide COVID-19 lockdown (difference between pre-confinement Barthel score and post-confinement Barthel score) for each sex.

These changes in the Barthel scale score due to the COVID-19 pandemic confinement were similar in both sexes, Figures 3, 4. No significant differences were observed between sexes with respect to Barthel index score before confinement (*z* = −0.217; *p* = 0.828), after confinement (*z* = −0.269; *p* = 0.788), or as a consequence of changes during confinement (*z* = −0.269; *p* = 0.788).

Pooling the data for the period 2020–2023, before confinement, the mean Barthel index score was 43.28 ± 19.017. During the COVID-19 pandemic, confinement functional capacity improved (*t* = −5.823; *p* < 0.001), with a mean Barthel index score of 54.41 ± 26.115 in June 2020. From this date until June 2023, a worsening in functional capacity was observed (*t* = 4.930; *p* < 0.001). Despite this worsening, the mean Barthel scale score in June 2023 (48.36 ± 21.711) remained higher than the pre-pandemic baseline score but without statistical significance (*t* = 0.555; *p* = 0.581), with a sawtooth observed in this period with peak improvement in June 2020.

### Aging as a modulator of functional response during COVID-19 pandemic confinement

Improvement in functional abilities during confinement occurred in all age groups. By age group, 91.7% (*n* = 11) of those younger than 80 years (*n* = 12), 69.7% (*n* = 46) of those aged 80–89 years (*n* = 66), and 64.7% (*n* = 22) of those aged 90 years or older (*n* = 34) improved their ability to perform basic activities of daily living during the COVID-19 pandemic confinement. In contrast, 8.3% (*n* = 1) of those younger than 80 years, 30.3% (*n* = 20) of those aged 80–89 years, and 35.3% (*n* = 12) of those aged 90 years or older worsened their functional abilities during confinement. The remaining components of this cohort maintained their Barthel index scores. Although there was a tendency for improvement capacity to decrease with increasing age, the differences did not reach statistical significance (χ^2^ = 3.157; *p* = 0.206).

Analysis comparing age groups, establishing 80 and 90 years of age as cutoff points, did not show a greater improvement in functional capacity among persons under 80 years of age, with respect to those of that age or older (OR 5.254; CI 0.650–42.476). Nor was it observed among persons under 90 years of age for those who were that age or older (OR 1.481; CI 0.625–3.510).

### The housing situation factor as a modulator of functional response during COVID-19 pandemic confinement

The effect of the housing situation of the dependent persons was analyzed as a modulator of the response to the functional capacity of the COVID-19 pandemic home confinement. This analysis showed that 83.7% (*n* = 36) of the dependent persons living independently (*n* = 43) improved their functional capacity; meanwhile, 64% (*n* = 32) of those living with their children improved their functional capacity, and 57.9% (*n* = 11) of those living with people other than their children or partner (*n* = 19) showed improvement in their functional capacity. In contrast, 16.3% (*n* = 7) of those living independently, 36% (*n* = 18) of those living with their children, and 42.1% (*n* = 8) of those living with others worsened in their functional capacity (χ^2^ = 6.086; *p* = 0.048). The remaining dependent persons of this cohort did not modify their Barthel index score.

Grouping housing situations between living independently or living with others, including adult children and dependent persons who, during the COVID-19 pandemic confinement, lived alone or with a partner, showed greater improvement in the ability to perform basic activities of daily living than those who lived with their children or with others (*z* = −2.054; *p* = 0.040). Dependent persons were more likely to respond to confinement by increasing functional capacity when living independently (OR 3.110; CI 1.209–7.999).

### Survival of the functionally dependent population of the orcasitas cohort from 2020 to 2023

In the follow-up of this cohort after the end of the nationwide COVID-19 lockdown in June 2020, it was observed that in June 2021, survival was 80.3%, with 19.7% of members dying during this year; at the end of the 3-year follow-up period (June 2023), 40.9% of the members of the Orcasitas cohort had died. This outcome affected people with Barthel's < 20 to a greater extent [78.95% (*n* = 15)]. Three-year mortality was not associated with BMI, marital status, economic or educational level, or the burden of chronicity (Tables 1, 4).

**Table 4:** Clinical and sociodemographic data of the Orcasitas cohort in June 2020 and analysis of their relationship with survival in June 2023.

**Sociodemographic data orcasitas cohort and survival in June 2023**			
	**Value**	**Statistical**	**Significance**
**Survival**, ***n*** **(%)**			
Alive	75 (59.05%)	–	–
Deceased	52 (40.95%)		
**Sex**, ***n*** **(%)**			
Male	27 (21.3%)	χ^2^ = 6.875	*p* = 0.009
Female	100 (78.7%)		
**Clinical variables**			
Age (mean ± SD)	86.6 ± 6.3	*z*: −0.046	*p* = 0.964
BMI (mean ± SD)	28.58 ± 4.70	*z*: −0.238	*p* = 0.812
Barthel pre-pandemic (mean ± SD):	43.28 ± 19.01	*z* = −3.646	*p* < 0.001
Barthel post-pandemic (mean ± SD):	54.41 ± 26.11	*z* = −4.313	*p* < 0.001
**Marital status**^a^, ***n*** **(%)**			
Married	39 (30.7%)	χ^2^ = 0.593^a^	*p* = 0.441
Not married^b^	88 (69.3%)		
**Education level**^c^, ***n*** **(%)**			
Insufficient^d^ (without education)	111 (88.1%)	χ^2^ = 4.531^b^	*p* = 0.033
Has education^e^	15 (11.9%)		
**Income level**, ***n*** **(%)**			
<11.200 euros/year	60 (47.24%)	χ^2^ = 7.219	*p* = 0.007
≥11.200 euros/year	67 (52.76%)		
**Chronicity burden**, ***n*** **(%)**			
≥5 chronic diseases e-SOAP^f^	34 (26.8%)	χ^2^ = 0.718	*p* = 0.397
<5 chronic diseases e-SOAP	92 (72.4%)		
**Polymedication**^g^ ***n*** **(%)**			
Yes	115 (90.6%)	χ^2^ = 2.488	*p* = 0.115
No	11 (8.7%)		

^a^Analysis of the differences between being married and having other marital statuses.

^b^Not married: includes widowed, separated, divorced, and single people.

^c^Analysis of the differences between having and not having an education.

^d^Includes: illiterate, has no schooling but can read and write, and not finished primary education.

^e^Includes having some type of education (primary, graduated, higher education).

^f^e-SOAP: IT tool that manages the Primary Care scorecard.

^g^Polymedicated: cutoff point 5 or more drugs or active substances.

This group was very old, and to avoid possible biases, only the group of people aged 80 years or older, representing 90.5% of this cohort, was included compared to the general non-dependent population. In relation to this age group, 36.7% (*n* = 33) of the dependent women (*n* = 90) and 60% (*n* = 15) of the men (*n* = 25) died in the period 2020–2023. Data from the Health System's IHC application shows 1,682 non-dependent persons in June 2020, 1,110 women and 572 men. In June 2023, 10.18% (113) of women and 9.6% (55) of non-dependent men were no longer registered in this application, with 997 (92.6%) women and 517 (92.8%) men remaining registered. Compared to the non-dependent population, the functionally dependent population aged 80 years or older in this cohort was more likely to drop out of the registries in the following 3 years (OR 5.767; CI 3.882–8.568), a situation that occurred in both sexes, women (OR 5.108; CI 3.190–8.179) and men (OR 11.750; CI 5.235–26.370).

When the data were analyzed using the classic classification of dependence into four levels, a method used in the Spanish National Health System, it was observed that the risk of mortality increased progressively as the level of dependence increased until it became significant between mild dependence and total dependence. The difference between mild dependence and moderate or severe dependence was not significant (Table 5). The difference between mild dependence and moderate- and severe-dependence groups was not significant (Table 5). One year after the end of the nationwide COVID-19 lockdown, the pre-pandemic level on the Barthel scale was not associated with survival, but the post-pandemic level was (Table 6). Three years after the end of confinement, both the pre-pandemic level and the post-pandemic level were associated with survival (Table 6), with higher mortality observed among people with severe and total functional dependence.

**Table 5:** Survival at 1 year and 3 years of follow-up among people with functional dependence in the Orcasitas cohort in relation to their level of dependence and the changes in the Barthel index during the nationwide COVID-19 lockdown decreed by the government of Spain.

**Functional dependence in basic activities of daily living and survival in the period 2020–2023**						
**Survival June 2021**						
**Variable** ***n*** **(%)**			**Data**	**HR**	**95% CI**	**Significance**
Classic Barthel dependency level^b^	Mild (Barthel 60)^a^:		43 (33.8%)	–	–	–
	Moderate (Barthel 20–35)		46 (36.2%)	1.309	0.449–3.814	*p* = 0.622
	Severe (Barthel 40–55)		19 (15%)	2.716	0.944–7.814	*p* = 0.064
	Total (Barthel < 20)		19 (15%)	3.168	1.151–8.723	*p* = 0.026
Barthel dependency level cutoff point 40^b^	Severe	Alive	25 (65.79%)	0.498	0.203–1.222	*p* = 0.128
		Deceased	13 (34.21%)			
	Moderate	Alive	77 (86.52%)			
		Deceased	12 (13.48%)			
Barthel score change due to confinement^b^ (*n* = 112)	Improved^c^	Alive	64 (57.14%)	1.347	0.383–4.743	*p* = 0.643
		Deceased	15 (13.39%)			
	Worsened^d^	Alive	26 (23.21%)			
		Deceased	7 (6.25%)			
**Survival June 2023**						
**Variable** ***n*** **(%)**			**Data**	**HR**	**95% CI**	**Significance**
Classic Barthel dependency level^b^	Mild (Barthel 60)^a^:		43 (33.8%)	–	–	–
	Moderate (Barthel 20–35)		46 (36.2%)	1.168	0.619–2.205	*p* = 0.631
	Severe (Barthel 40–55)		19 (15%)	1.886	0.992–3.585	*p* = 0.053
	Total (Barthel < 20)		19 (15%)	2.829	1.659–4.823	*P* < 0.001
Barthel dependency level cutoff point 40^b^	Severe	Alive	13 (34.21%)	0.718	0.598–0.861	*P* < 0.001
		Deceased	25 (65.79%)			
	Moderate	Alive	62 (69.66%)			
		Deceased	27 (30.34%)			
Barthel score change due to confinement^b^(*n* = 112)	Improved^c^	Alive	50 (44.64%)	1.077	0.447–2.597	*p* = 0.868
		Deceased	29 (25.89%)			
	Worsened^d^	Alive	16 (14.29%)			
		Deceased	17 (15.18%)			

^a^The contrast was carried out against the level of mild dependence.

^b^HR, hazard ratio, univariate regression analysis.

^c^HR, hazard ratio, multivariate regression analysis, adjusted for sex, educational level, and economic level.

^d^Patients' scores on the Barthel index improved after home confinement.

^e^Patients' scores on the Barthel index worsened after home confinement.

**Table 6:** Evolution of functional dependence during COVID-19 pandemic home confinement, estimated using the Barthel index, and its relationship with survival at 1 year (June 2021) and 3 years (June 2023).

**Barthel index, nationwide COVID-19 lockdown, and survival**				
**Survival in June 2021**				
**Barthel pre-pandemic level**				
**Variable**	**Status**	**Data** ***n*** **(%)**	**Statistical**	**Significance**
Total dependency (Barthel < 20)	Alive	12 (9.4%)	X^2^ = 7.584	*p* = 0.055
	Deceased	7 (5.5%)		
Severe dependency (Barthel 20–35)	Alive	13 (10.2%)		
	Deceased	6 (4.7%)		
Moderate dependency (Barthel 40–55)	Alive	39 (30.7%)		
	Deceased	7 (5.5%)		
Mild dependence (Barthel 60)	Alive	38 (29.9%)		
	Deceased	5 (5.5%)		
**Barthel post-confinement level**				
Total dependency (Barthel < 20)	Alive	8 (6.3%)	X^2^ = 11.734	*p* = 0.008
	Deceased	6 (4.7%)		
Severe dependency (Barthel 20–35)	Alive	16 (12.6%)		
	Deceased	3 (2.4%)		
Moderate dependency (Barthel 40–55)	Alive	18 (14.2%)		
	Deceased	9 (7.1%)		
Mild dependence (Barthel ≥ 60)	Alive	60 (47.2%)		
	Deceased	7 (5.5%)		
**Survival in June 2023**				
**Barthel pre-pandemic level**				
**Variable**	**Status**	**Data** ***n*****(%)**	**Statistical**	**Significance**
Total dependency (Barthel < 20)	Alive	4 (3.1%)	X^2^ = 16.766	*p* = 0.001
	Deceased	15 (11.8%)		
Severe dependency (Barthel 20–35)	Alive	9 (7.1%)		
	Deceased	10 (7.9%)		
Moderate dependency (Barthel 40–55)	Alive	31 (24.4%)		
	Deceased	15 (11.8%)		
Mild dependence (Barthel 60)	Alive	31 (24.4%)		
	Deceased	12 (9.4%)		
**Barthel post-confinement level**				
Total dependency (Barthel < 20)	Alive	2 (1.6%)	X^2^ = 19.976	*P* < 0.001
	Deceased	12 (9.4%)		
Severe dependency (Barthel 20–35)	Alive	10 (7.9%)		
	Deceased	9 (7.1%)		
Moderate dependency (Barthel 40–55)	Alive	13 (10.2%)		
	Deceased	14 (11%)		
Mild dependence (Barthel ≥ 60)	Alive	50 (39.4%)		
	Deceased	17 (13.4%)		

Taking a cutoff point on the Barthel index of 40 as a reference, in June 2021, 34.21% of people with severe dependency died, a percentage that rose to 65.79% in June 2023. Among people with moderate dependence, these percentages were 13.48 and 30.34%, respectively.

**Figure 5:**
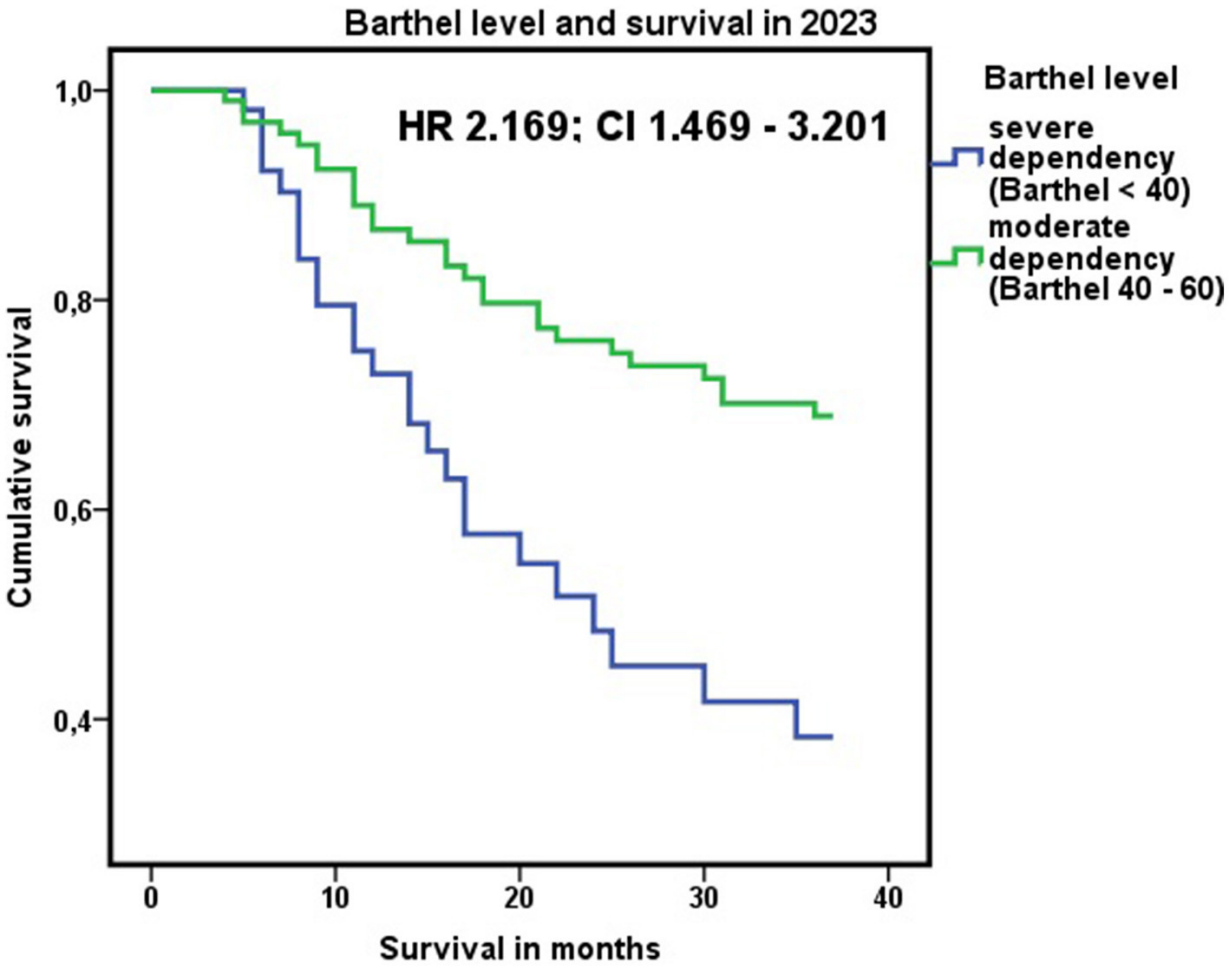
Association between the level of dependency (<40 or ≥40 on the Barthel index) and cumulative mortality through June 2023. HR 2.169; CI 1.469–3.201. Cox regression: survival in 2023 adjusted for age, sex, educational level, and income level of the dependent person.

A lower score on the Barthel index and a higher degree of dependence were associated with higher mortality (Tables 1, 4, 5). Patients with severe dependence had a greater probability of dying than patients with moderate dependence during this period, with an HR in June 2021 of 2,537 (CI 1.277–5.041) and 2.169 (CI 1.469–3.201) in June 2023. These results were maintained when the data were adjusted for age, sex, educational level, and income level, with an HR of 2.606 (CI 1.314–5.167) in June 2021 and an HR of 2.227 (CI 1.514–3.276) in June 2023, and by Cox regression. The results are represented graphically in Figure 5.

After confinement for the COVID-19 pandemic, 43.3% (*n* = 55) of the individuals in this cohort were no longer dependent. Of these, 74.5% (*n* = 41) were still alive at the end of the 3-year follow-up period, with the remaining 25.5% (*n* = 14) dying. Among those who remained dependent, 47.2% (*n* = 34) were still alive at 3 years, and 52.8% (*n* = 38) died. During the COVID-19 pandemic confinement, those dependent persons who improved their functional abilities to the point of no longer being considered dependent had a higher probability of survival at 3 years (OR 0.306; CI 0.142–0.655).

A cutoff point of 40 on the Barthel index was a better indicator of the risk of mortality associated with dependency than the classic classification into four levels (Table 5). No influence on survival was observed in 2021 or 2023, with an improved or worsened Barthel score between pre- and post-confinement. When this relationship was analyzed according to level of dependence and sex, however, women with moderate dependence who improved their Barthel score during confinement had a higher probability of survival at 3 years (OR 3.700; CI 1.111–12.327).

However, in Figure 1, the quadrant delimited by an age higher than the mean age and a Barthel index score lower than the cohort mean presented the highest mortality in the period 2020–2023.

In 2023, the mean age was 85.89 ± 5.63 years, and the mean BMI was 29.25 ± 4.33. In the 2020–2023 period, there was greater mortality in men than in women; 63% of the men and 35% of the women in the Orcasitas cohort died, with an HR for the male sex of 1.745 (CI 1.045–2.915) (Table 2). In 2023, 24.4% (*n* = 31) of initially functional-dependent people still had a Barthel score >60, thus falling outside the Health System dependency protocol.

### The Barthel index and the items that compose it as predictors of survival

**Figure 6:**
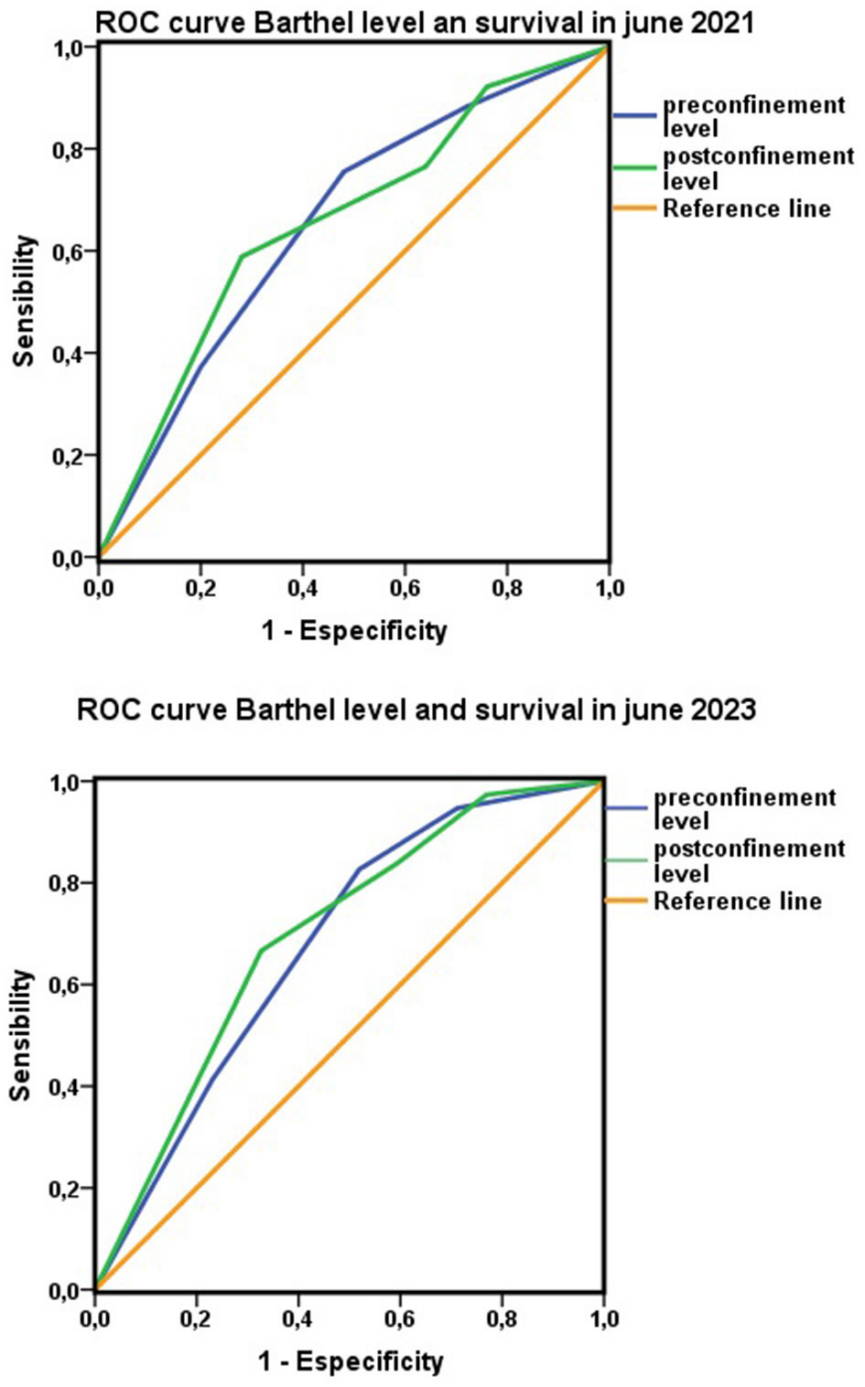
Barthel index and survival data. Areas under the ROC curve for levels on the Barthel index before and after nationwide COVID-19 lockdown with respect to survival in 2021 and 2023.

**Figure 7:**
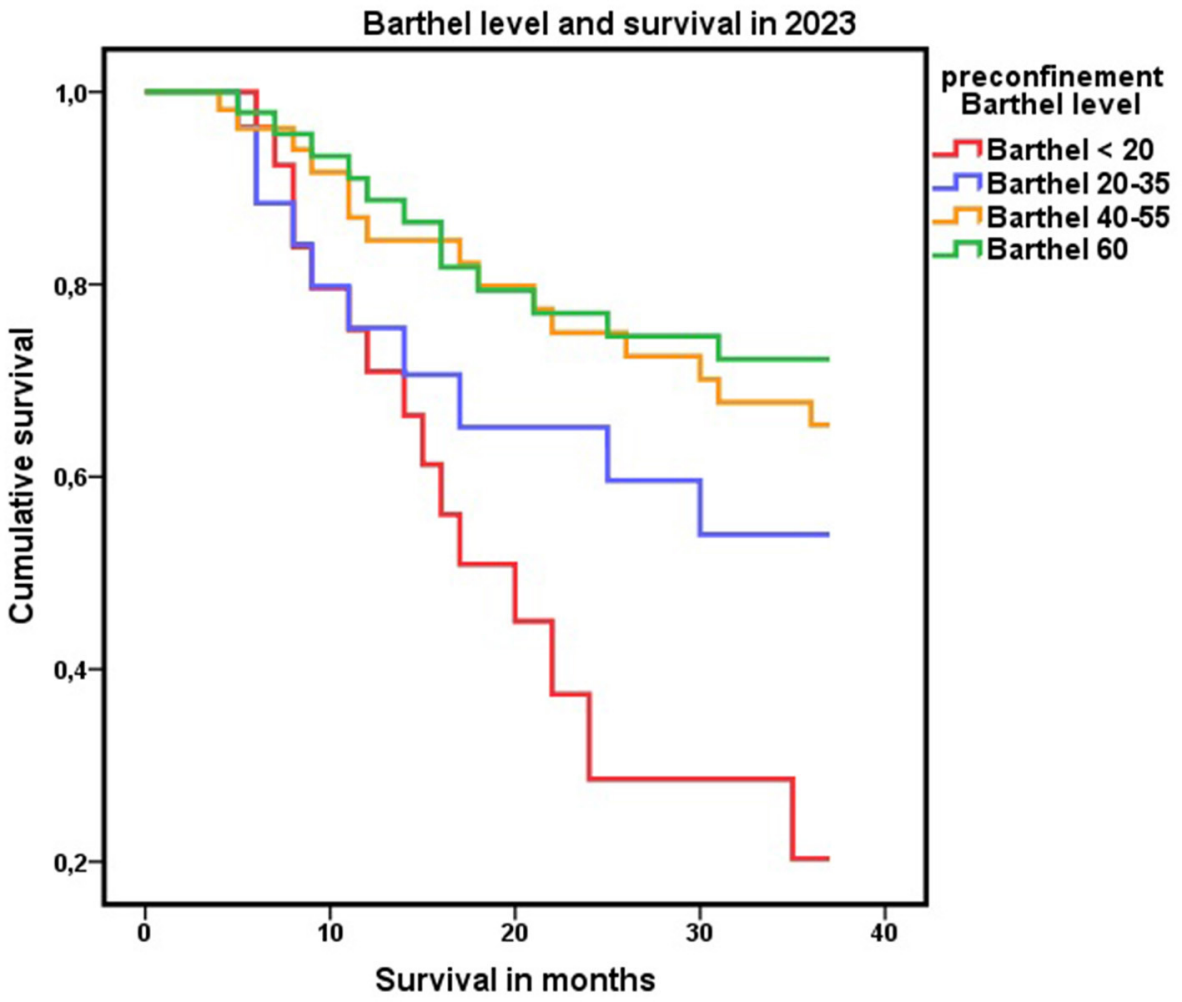
Level of dependence on the Barthel Index. Cox regression analysis of mortality at 3 years of follow-up according to the baseline level on the Barthel index.

Finally, when the ability of the Barthel index as a test to discriminate mortality associated with dependence was analyzed using ROC curves, both the score and the level on the Barthel index had confidence intervals above 0.5, indicating the existence of a discriminative capacity between persons with and without dependency. However, in both cases, the AUCs were between 0.5 and 0.7, indicating a low degree of discrimination (Figure 6). By Cox regression and adjusting for possible confounding factors, the Barthel index showed that at the cohort level, it could adequately discriminate population mortality risk (Figure 7).

**Figure 8:**
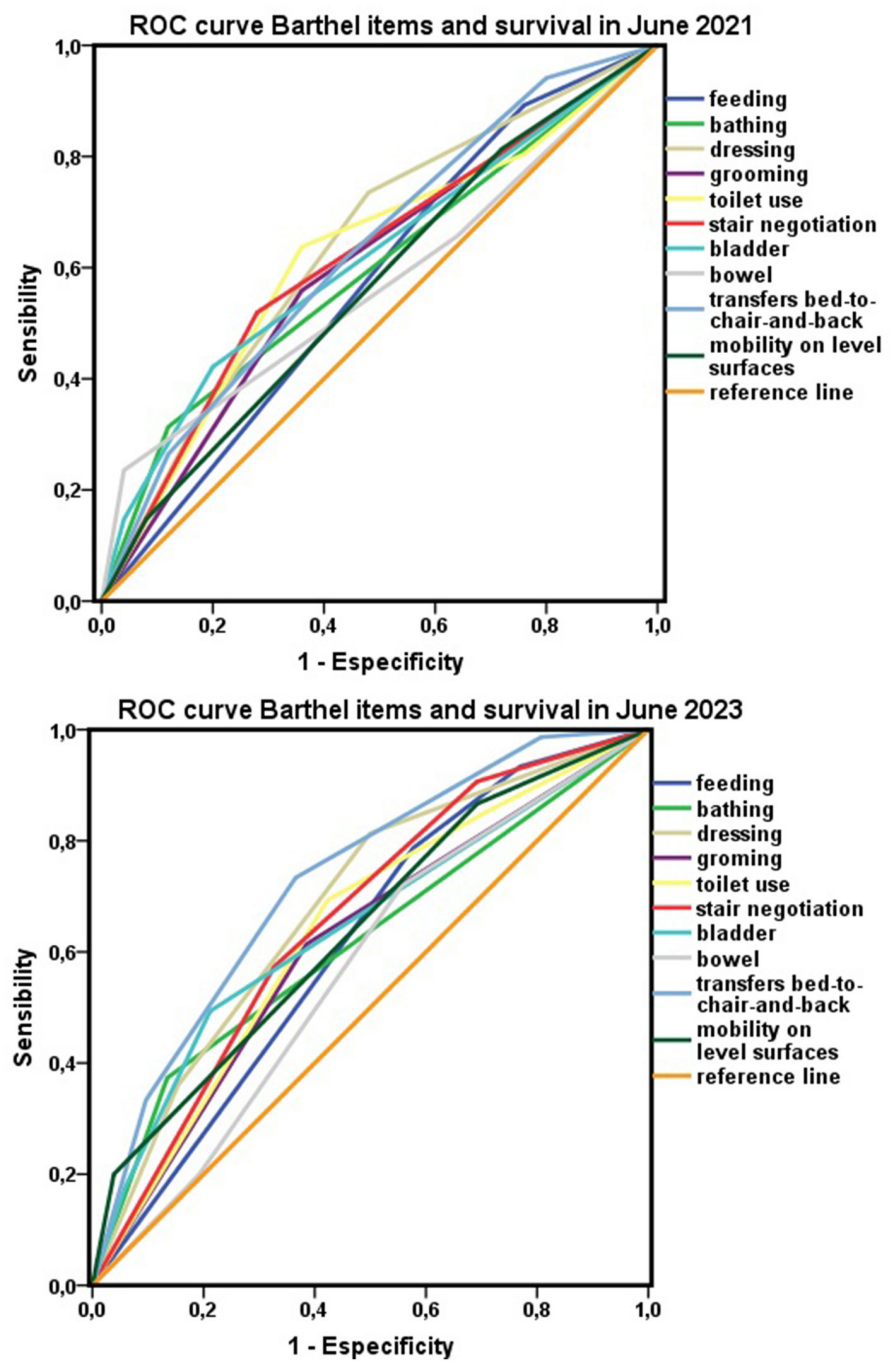
Items of the Barthel index and survival. Areas under the ROC curve for each item of the Barthel index with respect to survival in 2021 and 2023.

Breaking down the Barthel index according to its items to analyze the influence of each one in relation to survival, at 1 year, the discriminative capacity as a test of most of the items was low. However, the discriminative capacity of some items increased over time. For example, during follow-up, the AUC of the Barthel item “chair–bed transfers” improved to the category “useful in some circumstances” in 2023 (Figure 8).

## Discussion

The COVID-19 pandemic was the reason for carrying out this study, which made visible an already pre-existing situation, that of a population that was to a certain extent invisible, with a high mortality rate, and about which there was a great lack of knowledge. This lack of knowledge led to the prediction of results that reality showed to be radically different. The confinement and the risk of death due to the pandemic produced an awakening in the functionally dependent population of the Orcasitas cohort as a form of resilience in the face of adversity. This awakening, in response to the challenge posed to the functionally dependent population by being locked in their homes for months at a time, was observed across all ages and showed a non-significant tendency to become less frequent as the group analyzed got older.

The way in which older people lived in their environment influenced the level of functional improvement observed. A significant trend toward greater functional improvement was found among people living alone or with a partner, compared to those living with their children or others. Having fewer social support resources forced these people to respond to adversity in a way that people who did have these resources may not have needed since their cohabitants covered their basic subsistence needs.

In addition to making it possible to visualize a vulnerable group due to their frailty, this study provided relevant results on a diagnostic tool that is easy to use and inexpensive, the Barthel index. This tool showed its full potential as a predictor of short-term mortality during the COVID-19 pandemic (2, 18–20, 24–28). In this study, it was also a good predictor of medium- and long-term mortality (27–29). These results place the Barthel index at an advantage as a tool for detecting at-risk populations in health planning (20, 27–29) and in risk assessment in multiple clinical situations requiring discriminating the risk of adverse events.

In older persons, functional dependence is associated with greater mortality (24, 25), and this study showed that this risk was significantly higher in men than in women and that it was greater the higher the level of dependence (26, 27). These results are similar to those reported in other studies and show that age and sex influence the development and progression of functional disability (28). However, sex, age, and dependence are not isolated factors; rather, they evolve in a context that, as other studies have also reported, influences the observed results (29). The context described in this cohort is an unfavorable social environment, consisting of a suburb of the capital of Spain with an aging population, in which one in five people is 65 or older, and with a large group of people over 80 years. This environment was complemented by unfavorable economic and cultural indicators. The population with functional dependence in the Orcasitas cohort had a low socioeconomic level, which placed almost half of them in the vicinity of the poverty threshold; in 2020, the poverty threshold in Spain was 9,626 euros/year. In addition, they shared a low cultural level, with almost all the dependents in this cohort having an insufficient level of education.

It is in this social environment that the nationwide confinement by COVID-19 took place, with an unexpected effect observed in the population with functional dependence due to this confinement: improvement in functional capacity to perform basic activities of daily living. Although initially this response could be considered a temporary adaptive response to a stressful event that put survival at risk, longitudinal follow-up showed that it involved greater changes. Changes were observed not only in the persistence of improved functional dependence at 3 years but also in that many people ceased to be dependent after confinement and maintained and regained functional independence in the long term. Such changes were relevant in terms of survival.

The presence of a very old dependent population that potentially has few cultural and economic coping tools in an environment of home confinement was expected to worsen baseline conditions (30–32), which occurred during the pandemic in institutionalized patients (33–36). However, in the non-institutionalized dependent population of the Orcasitas cohort, nationwide COVID-19 lockdown triggered an “awakening” phenomenon, developing survival defense mechanisms that made it possible to improve their functional capacities, with a direct effect: half of the dependent persons in this cohort ceased to be dependent. This result was also observed in people with total dependence, in a more moderate form and in relation to their important limitations. This change left a visible imprint as a sawtooth in the functional assessment conducted with the Barthel index between 2020 and 2023. Research on the pandemic and home confinement has shown that dependency is a dynamic process that is susceptible to intervention (37), and that there is no age limit for this intervention.

In addition to the data described above on the social environment, the population of Orcasitas came from a socioeconomic immigration background to the outskirts of large cities in the middle of the last century and lived for many years in substandard housing or shantytowns. These individuals were the first marginal population group to design their own housing for rehousing in the 1970s and 1980s, forming a social group with a markedly vindictive nature that may have generated an important resilience in the face of adversity (37, 38), which could justify the improvement described. On the other hand, the loss of support resources, such as daycare centers or home-help assistants, forced them to carry out activities that they had not previously done, a situation that has been described as increasing resilience (39) and which did not happen in nursing homes (40).

The intervention of protective psychosocial factors acquired prior to exposure to the new stressor posed by the COVID-19 pandemic has already been described in other studies (41) and probably played a relevant role in the fact that the results observed in this study did not describe functional deterioration. This deterioration is usually the predictable outcome when people with frailty are subjected to prolonged stressors and was observed during COVID-19 pandemic confinement in institutionalized individuals or those requiring hospital admissions, including admissions for COVID-19 (2, 27, 32–35, 42, 43). These living situations (nursing home, hospital) generally entail an added dependence on third parties (nursing home staff, health professionals), which can affect the person's own self-concept (43) and the innate resilience or resilience developed over the course of life, making its protective effect less visible or non-existent (44).

The increase in life expectancy has led to the appearance of new factors, which may have influenced the results observed in this study. These factors, such as subjective age (the age the patient feels he or she is), subjective aging (how the individual perceives his or her own aging process), and, in age groups as advanced as this one, the role of the subjective proximity of death (45, 46) probably play a role in the life history observed.

However, the data obtained also show that not all the dependent population behaved in the same way in the face of the stressor event of the COVID-19 pandemic, and these differences could be based on Antonovsky's theory of salutogenesis (47, 48), with implications in relation to survival. Beyond the classic coping strategies, part of this population, due to their origins, their life history (poverty at birth, emigration, living in substandard housing, unemployment, low or no education, low income), and their constant struggle to get by, would have developed a chronic adaptive resilience—resilience that they applied daily to survive in permanent adverse living conditions. This vital evolution would have generated psychosocial resources and even biological and material resources not studied (subsistence economy), which would be included within the so-called General Resilience Resources of Antonovsky and would have ended up giving a Sense of Coherence to their lives even in adversity. These resources would remain active or latent, depending on the person's basal state and degree of dependence, and the COVID-19 pandemic brought them to light. Its effect would be reflected in the person's capacity to make the necessary changes to improve his or her functionality, trying to survive the pandemic, changes that were recorded in this study employing the Barthel index, and for which age was not a limiting factor. However, this response showed that these changes had a result not only in terms of functionality but also in terms of survival, so those who made the changes were more likely to stay alive in the long term. This situation was observed in this study not only among women with moderate dependency, who improved their functional capacity during confinement and significantly increased their survival, but also in the observation that those who had the capacity to make positive changes and did make them, had a higher representation among those who survived to 3 years.

Functional dependence is a progressive process clearly associated with high mortality. The COVID-19 pandemic was relevant enough to motivate these people to face it within the “sense of coherence” proposed by Antonovsky. These people, despite their dependence, had the capacity to rearrange themselves again to survive. This capacity for improvement was independent of age, previous disease burden, and the presence of polypharmacy and was observed at all levels of functional dependence. These changes not only had short-term but also long-term effects. For instance, it implied that people who were functionally dependent before the pandemic ceased to be so with the pandemic and maintained this change over time, some of them being functionally independent 3 years later. All of this was in a context with a mean age of 86 years.

This functional improvement at the cohort level allowed them to gain time as the group currently has slightly better functionality than in 2020, even though almost half of the cohort died in this triennium.

In this line, the mean age of this cohort was advanced, and a high abandonment rate was to be expected due to the mere fact of natural deaths due to age (at the end of the study, the mean age, if no one had died, would have been 89 years, well-above life expectancy in Spain). However, among the non-dependent population over 80 years of age, the same abandonment rate was not observed, so being over 80 years of age and being functionally dependent was probably a risk factor for mortality. This cannot be confirmed in this study because the abandonment rate in the non-dependent population includes both deaths and changes of residence outside the Orcasitas neighborhood.

Mortality was higher in men, and although at the general population level, men have a lower life expectancy than women (49), other causes cannot be ruled out for the different mortality by gender observed in the dependent population. As expected, mortality was also higher in persons with greater functional dependence.

For non-institutionalized dependent patients, being included as a total dependent is equivalent to being placed on the threshold of death. The final causes of this mortality, its evolutionary course, and the actions developed by the different agents involved in its follow-up have been the subject of few studies. Moreover, this high mortality rate is not usually made visible by society, which associates it with aging itself, favoring ageism that increases social inequalities (7).

Although the percentage of non-institutionalized older persons with functional dependence might not seem to be high compared to the general population of the same age, the high mortality rate observed in this group makes it advisable to implement changes in health planning to address this problem. This problem, although not strictly health-related, form part of the holistic care that Primary Health Care should provide citizens to maintain equity in care. Above all, an environment where the aging of the population is likely to increase in functional dependence, justifies these actions.

Suggested changes to the current model of care for older persons with functional dependence are supported by the evidence observed in this study that there is room for improvement, there is no age limit for improvement, and this improvement influences long-term survival. Other studies also observe positive changes in long-term functional dependence through proactive actions in these persons (50).

The Barthel index is a simple method of assessment and, as confirmed by this study, it allows easy discrimination of population risk (19, 26, 42), being a good predictor of medium- and long-term mortality (27, 51, 52) and justifying its use to discriminate at-risk populations in health planning (19, 26, 41). In this study, we used two methods for assessing the Barthel index: the classic method, which consists of four levels of dependence, and the method that establishes two levels of dependence, with a cutoff point at Barthel 40. The justification for this approach is 2-fold: on the one hand, to favor comparison of the results of this study with those of other studies, and, on the other hand, to allow health planning to benefit from this double criterion to establish the appropriate indicator in relation to survival.

This study unified the risk levels “total dependence” and “severe dependence” into a single level of severe dependence with a cutoff point of 40 on the Barthel index. This showed greater discrimination in terms of the risk of death. This result, supported by the guidelines of some health systems (23, 51, 53), allows us to recommend using this criterion in medium- and long-term health planning to obtain better health results. The discriminative power of the Barthel index as a test seems to increase as the cohort follow-up is prolonged, as in parallel, each of the items that make up the scale.

A limitation of this study is that the sample size and follow-up time did not allow us to reach greater statistical significance. This could perhaps be present in larger samples or over longer times. Another limitation of the study is that non-parametric estimates of the AUC tend to be underestimated when data from discrete rating scales are used, such as those included in the Barthel index (54).

Evaluating the Barthel index, the pre-confinement level prior to March 2020 was taken as a reference to avoid the biases of the pandemic, the loss of attendant care for domestic work, and the high variability of care of non-cohabitating children toward their parents in this phase. These factors caused a “storm” among dependents, and their effect on the items comprising the Barthel index was not predictable.

The time variable had its effect on this baseline state, “filtering” in terms of survival of those items that are most related to it and highlighting those on which health planning should have an impact to minimize its impact and improve the quality of life of these people. As the components of this population died, this filter effect on the time variable showed its potential, allowing us to detect new questions about the indisputable value of the Barthel index, such as the extent to which the loss of mobility is the factor that leads to this higher mortality among people with functional dependence.

The role of each item of the Barthel index in identifying the most vulnerable patients should be established. Furthermore, items that assess instrumental ADLs should be integrated to provide more individualized care to provide equivalent care for non-institutionalized people with dependency and those in institutionalized care (54).

In summary, unexpected confinement that was forced on this cohort did not have the same effect on functional deterioration as that produced by other sudden events, such as hospital admission (3, 18) or institutionalization (16). Nor was it associated with progressive deterioration associated with age and comorbidities (9, 10), social isolation (20, 27, 55), or the situation of loneliness that affects many of our older people (56). A stressful situation, such as a pandemic, triggers response mechanisms to ensure survival—mechanisms that are present in all individuals, regardless of their level of dependence (35). The results for the Orcasitas cohort, in line with what is proposed in Antonovsky's theory of salutogenesis (57, 58), indicate a resilient population that shows strength in an unforeseen situation and attempts to give the best of itself to be able to move forward (38). Longitudinal studies are needed to analyze the characteristics of the non-institutionalized functionally dependent population and establish policies to improve the quality of life of this population group (54, 59) and to move away from the stereotypical image of dependent persons who consume many resources without contributing anything, promoting their integration in the face of growing social discrimination based on ageism (60).

## Conclusions

The functionally dependent population that is not institutionalized in nursing homes and remains in the community is a group that is, to some extent, invisible in epidemiological studies. Advanced age, and especially very advanced age, such as that of this cohort, predisposes us to assimilate that mortality is part of the normal evolutionary course. Similarly, we assimilate the loss of functional capacities as normal. However, accepting these premises should not imply doing nothing to improve the quality of life of these people. Perhaps the main result of this study has been to show that there is a capacity for improvement at any age. And that this capacity for improvement is maintained at all levels of functional dependence. Stereotypes and labels can be a source of inequalities. Against ageism, integration, the promotion of the maintenance of social roles, and the development of activities that encourage the maintenance or recovery of functional capacities may be the appropriate strategies to maintain equity in the care of our older people.

## Data availability statement

The original contributions presented in the study are included in the article/Supplementary material, further inquiries can be directed to the corresponding author.

## Ethics statement

The studies involving humans were approved by Center Local Research Commission of the Primary Care Management of Madrid (reference number 16/20-C-Bis) on June 29, 2020. The Ethics Committee of the Hospital provided approval by resolution CEIm 23/501 dated September 26, 2023. The studies were conducted in accordance with the local legislation and institutional requirements. Written informed consent for participation in this study was provided by the participants' legal guardians/next of kin.

## Author contributions

VM: Conceptualization, Data curation, Formal analysis, Funding acquisition, Investigation, Methodology, Project administration, Resources, Software, Supervision, Validation, Visualization, Writing – original draft, Writing – review & editing. MM: Conceptualization, Data curation, Formal analysis, Investigation, Methodology, Project administration, Software, Supervision, Validation, Visualization, Writing – original draft, Writing – review & editing. MF: Conceptualization, Data curation, Formal analysis, Investigation, Methodology, Project administration, Software, Supervision, Validation, Visualization, Writing – original draft, Writing – review & editing. AM: Conceptualization, Data curation, Formal analysis, Investigation, Methodology, Project administration, Software, Supervision, Validation, Visualization, Writing – original draft, Writing – review & editing. MB: Conceptualization, Data curation, Investigation, Methodology, Project administration, Software, Supervision, Validation, Visualization, Writing – original draft, Writing – review & editing. HA: Conceptualization, Formal analysis, Project administration, Supervision, Validation, Visualization, Writing – original draft, Writing – review & editing. EP: Conceptualization, Data curation, Investigation, Methodology, Project administration, Software, Supervision, Validation, Visualization, Writing – original draft, Writing – review & editing. LC: Conceptualization, Data curation, Investigation, Methodology, Project administration, Software, Supervision, Validation, Visualization, Writing – original draft, Writing – review & editing. SG: Conceptualization, Data curation, Formal analysis, Investigation, Methodology, Project administration, Software, Supervision, Validation, Visualization, Writing – original draft, Writing – review & editing. ESá: Conceptualization, Data curation, Formal analysis, Investigation, Methodology, Project administration, Software, Supervision, Validation, Visualization, Writing – original draft, Writing – review & editing. ESe: Conceptualization, Data curation, Formal analysis, Investigation, Methodology, Project administration, Software, Supervision, Validation, Visualization, Writing – original draft, Writing – review & editing. IS: Conceptualization, Data curation, Formal analysis, Investigation, Methodology, Project administration, Software, Supervision, Validation, Visualization, Writing – original draft, Writing – review & editing. MR: Conceptualization, Data curation, Formal analysis, Investigation, Methodology, Project administration, Software, Supervision, Validation, Visualization, Writing – original draft, Writing – review & editing. JH: Conceptualization, Data curation, Formal analysis, Investigation, Methodology, Project administration, Software, Supervision, Validation, Visualization, Writing – original draft, Writing – review & editing. IL: Conceptualization, Formal analysis, Methodology, Supervision, Validation, Visualization, Writing – original draft, Writing – review & editing.

## GIDO Collaborative Group (Orcasitas Dependency Research Group)

Vicente Martín Moreno, Orcasitas Health Care Center and i+12 Research Institute of the Doce de Octubre hospital; GIDO group co-director, Madrid, Spain; María Inmaculada Martínez Sanz, Orcasitas Health Care Center; GIDO group co-director, Madrid, Spain; Miriam Fernández Gallardo, María Palma Benítez Calderón, Helena Alonso Samperiz, Elena Pérez Rico, Laura Calderón Jiménez, Sara Guerra Maroto, Elena Sánchez Rodríguez, Eva Sevillano Fuentes, Irene Sánchez González, Julia Herranz Hernando, and Irene León Saiz, Orcasitas Health Care Center, Madrid, Spain; Amanda Martín Fernández, Polibea Concert, Madrid, Spain; Miguel Recuero Vázquez, Orcasitas Health Care Center and Nursing Home Care Unit of the Center Assistance Directorate, Madrid, Spain.
