# Supplementary material for: The influence of nationwide COVID-19 lockdown on the functional impairment and long-term survival of dependent people for carrying out basic activities of daily living in a neighborhood of the city of Madrid, Spain: Orcasitas Cohort Longitudinal Study
Source: Front Public Health. 2024 Jul 9;12:1385058. doi: 10.3389/fpubh.2024.1385058 (PMC11263189; doi:10.3389/fpubh.2024.1385058)
Supplement: Supplementary file 1 [file Table_1.DOCX]

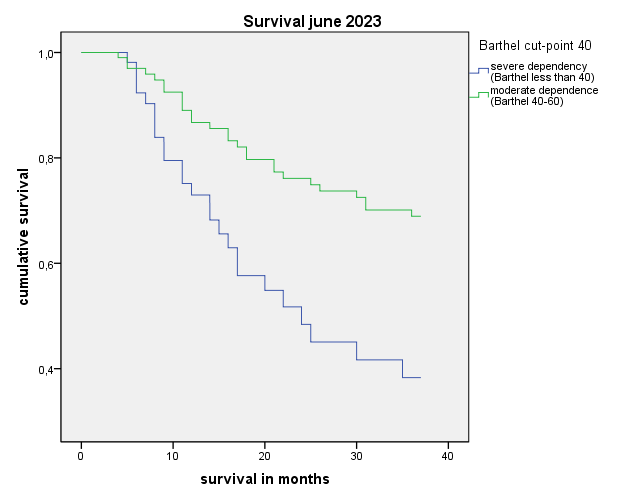


HR 2.169; CI 1.469-3.201

|  | | |
| --- | --- | --- |
|  | | |
| Chi-square | gl | Sig. |
| 18.242 | 4 | 0.001 |

|  | B | ET | Wald | gl | Sig. | Exp (B) | 95,0% CI for Exp(B) | |
| --- | --- | --- | --- | --- | --- | --- | --- | --- |
|  |  |  |  |  |  |  | Lower | Superior |
| Sex | -0.808 | 0.305 | 7.033 | 1 | 0.008 | 0.446 | 0.246 | 0.810 |
| Level of studies | -0.997 | 0.370 | 7.252 | 1 | 0.007 | 0.369 | 0.179 | 0.762 |
| Income level | 0.678 | 0.294 | 5.316 | 1 | 0.021 | 1.969 | 1.107 | 3.504 |
| Age | 0.028 | 0.021 | 1.877 | 1 | 0.171 | 1.029 | 0.988 | 1.072 |

Figure 4: Association between the level of dependency (<40 or ≥40 on the Barthel Index) and cumulative mortality through June 2023. HR 2.169; CI 1.469-3.201. Cox regression: Survival in 2023 adjusted for age, sex, educational level, and income level of the dependent person.


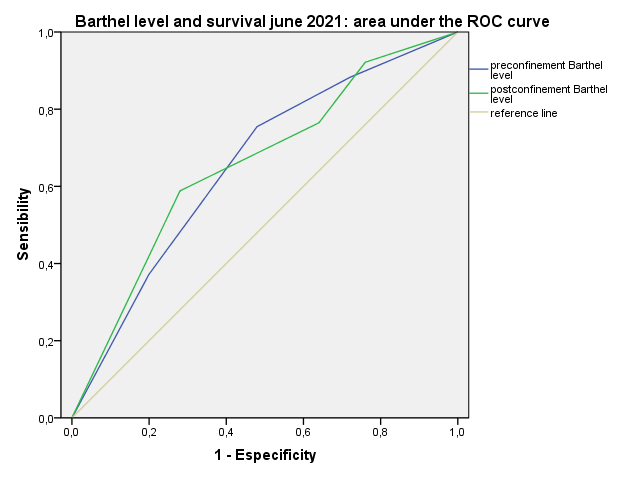


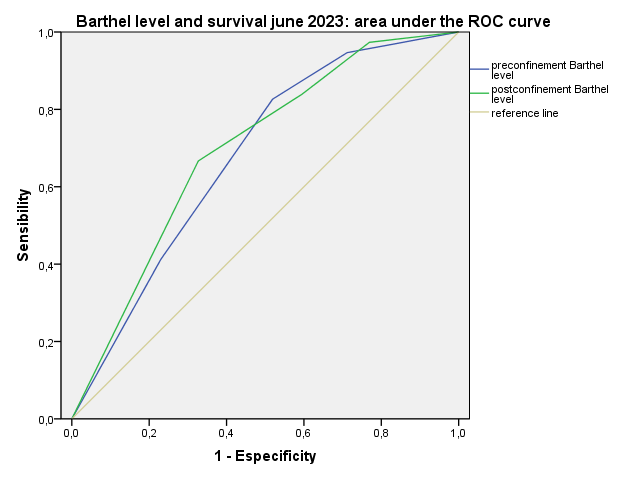


|  | | | | | |
| --- | --- | --- | --- | --- | --- |
| Variables | Area | ET | Sig. | 95,0% CI | |
|  |  |  |  | Lower | Superior |
| Preconfinement Barthel level | 0.655 | 0.063 | 0.016 | 0.532 | 0.779 |
| Postconfinement Barthel level | 0.658 | 0.061 | 0.015 | 0.539 | 0.777 |

|  | | | | | |
| --- | --- | --- | --- | --- | --- |
| Variables | Area | ET | Sig. | 95,0% CI | |
|  |  |  |  | Lower | Superior |
| preconfinement Barthel level | 0.678 | 0.050 | 0.001 | 0.580 | 0.775 |
| postconfinement Barthel level | 0.696 | 0.048 | 0.000 | 0.602 | 0.791 |

Figure 5: Barthel Index and survival data. Areas under the ROC curve for levels on the Barthel Index before and after nationwide COVID-19 lockdown with respect to survival in 2021 and 2023.


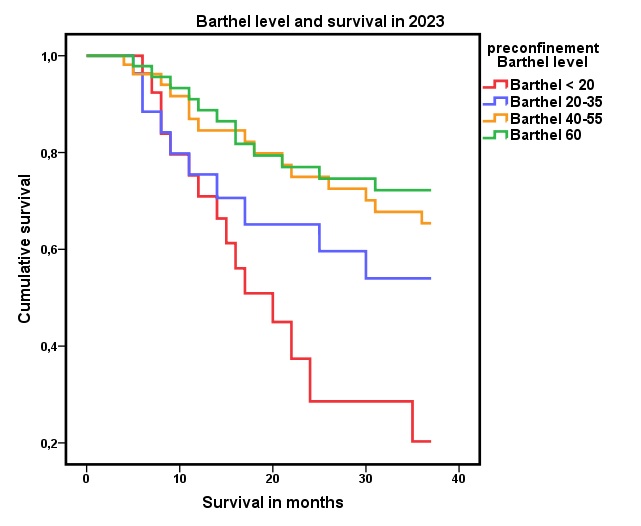


| Chi-square | gl | Sig. |
| --- | --- | --- |
| 20,806 | 4 | 0,0001 |

|  | | | | | | |  |  |
| --- | --- | --- | --- | --- | --- | --- | --- | --- |
|  | B | ET | Wald | gl | Sig. | Exp(B) | 95,0% CI for Exp(B) | |
|  |  |  |  |  |  |  | Lower | Superior |
| Sex | -0.699 | 0.318 | 4.837 | 1 | 0.028 | 0.497 | 0.266 | 0.927 |
| Income level | -0.705 | 0.296 | 5.662 | 1 | 0.017 | 0.494 | 0.276 | 0.883 |
| Age | 0.027 | 0.021 | 1.630 | 1 | 0.202 | 1.027 | 0.986 | 1.071 |
| Level of studies | 1.124 | 0.387 | 8.457 | 1 | 0.004 | 3.078 | 1.443 | 6.568 |

Figure 6: Level of dependence on the Barthel Index. Cox regression analysis of mortality at 3 years of follow-up according to the baseline level on the Barthel Index.


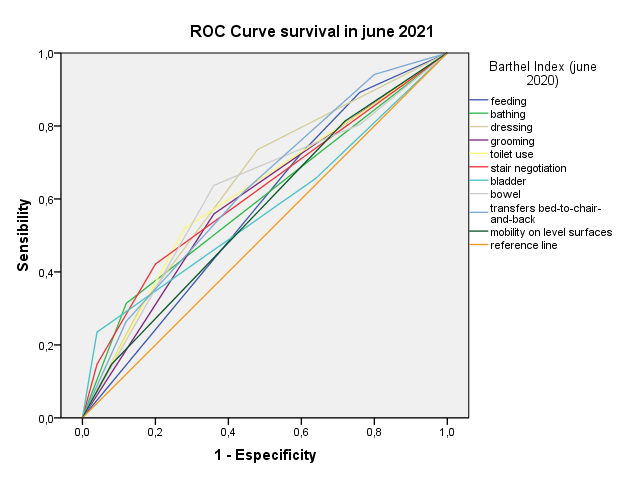


| **Area under the curve (AUC)** | | | | | |
| --- | --- | --- | --- | --- | --- |
| Test Result Variables (Barthel Index) | Area | Std. Error | Asymptotic significance | 95% CI | |
|  |  |  |  | Lower | Superior |
| feeding | 0.574 | 0.067 | 0.252 | 0.442 | 0.706 |
| bathing | 0.597 | 0.059 | 0.134 | 0.481 | 0.712 |
| dressing | 0.642 | 0.062 | 0.028 | 0.519 | 0.764 |
| grooming | 0.599 | 0.063 | 0.124 | 0.476 | 0.722 |
| toilet use | 0.617 | 0.061 | 0.069 | 0.498 | 0.736 |
| stair negotiation | 0.617 | 0.058 | 0.070 | 0.503 | 0.731 |
| bladder | 0.571 | 0.057 | 0.275 | 0.458 | 0.683 |
| bowel | 0.619 | 0.061 | 0.065 | 0.500 | 0.739 |
| transfers bed-to-chair-and-back | 0.632 | 0.063 | 0.042 | 0.509 | 0.755 |
| mobility on level surfaces | 0.567 | 0.064 | 0.298 | 0.442 | 0.692 |


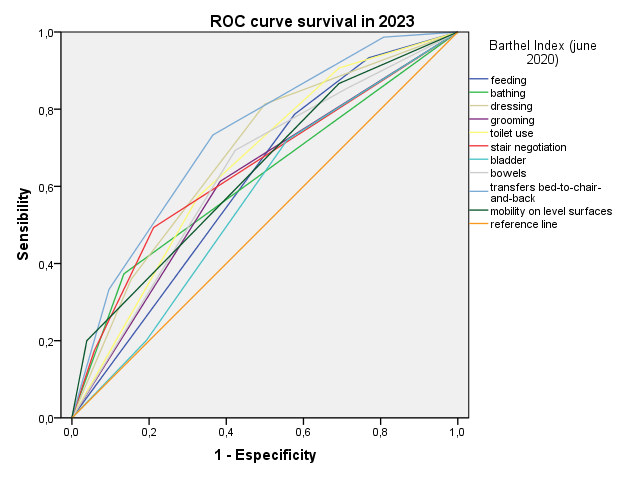


| **Area under the curve (AUC)** | | | | | |
| --- | --- | --- | --- | --- | --- |
| Test Result Variables (Barthel Index) | Area | Std. Error | Asymptotic significance | 95% CI | |
|  |  |  |  | Lower | Superior |
| feeding | 0.615 | 0.052 | 0.027 | 0.514 | 0.717 |
| bathing | 0.619 | 0.050 | 0.022 | 0.522 | 0.717 |
| dressing | 0.684 | 0.048 | 0.000 | 0.589 | 0.779 |
| grooming | 0.614 | 0.051 | 0.029 | 0.515 | 0.714 |
| toilet use | 0.657 | 0.050 | 0.003 | 0.559 | 0.756 |
| stair negotiation | 0.645 | 0.049 | 0.006 | 0.549 | 0.741 |
| bladder | 0.568 | 0.053 | 0.196 | 0.464 | 0.671 |
| bowel | 0.637 | 0.051 | 0.009 | 0.538 | 0.736 |
| transfers bed-to-chair-and-back | 0.731 | 0.045 | 0.000 | 0.642 | 0.820 |
| mobility on level surfaces | 0.640 | 0.049 | 0.008 | 0.543 | 0.736 |

Figure 7: Items of the Barthel Index and survival. Areas under the ROC curve for each item of the Barthel Index with respect to survival in 2021 and 2023.
